# Predictive models for diabetes mellitus using machine learning techniques

**DOI:** 10.1186/s12902-019-0436-6

**Published:** 2019-10-15

**Authors:** Hang Lai, Huaxiong Huang, Karim Keshavjee, Aziz Guergachi, Xin Gao

**Affiliations:** 1 Department of Mathematics and Statistics, York University, 4700 Keele Street, Toronto, Ontario M3J 1P3 Canada; 2 The Fields Institute for Research in Mathematical Sciences, Center for Quantitative Analysis and Modelling (CQAM) Lab, 222 College Street, Toronto, Ontario M5T 3J1 Canada; 3 Institute of Health Policy, Management and Evaluation, University of Toronto, 155 College Street, Suite 425, Toronto, Ontario M5T 3M6 Canada; 4 Ted Rogers School of Management - Information Technology Management, Ryerson University, 350 Victoria Street, Toronto, Ontario M5B 2K3 Canada

**Keywords:** Diabetes mellitus, Machine learning, Gradient boosting machine, Predictive models, Misclassification cost

## Abstract

**Background:**

Diabetes Mellitus is an increasingly prevalent chronic disease characterized by the body’s inability to metabolize glucose. The objective of this study was to build an effective predictive model with high sensitivity and selectivity to better identify Canadian patients at risk of having Diabetes Mellitus based on patient demographic data and the laboratory results during their visits to medical facilities.

**Methods:**

Using the most recent records of 13,309 Canadian patients aged between 18 and 90 years, along with their laboratory information (age, sex, fasting blood glucose, body mass index, high-density lipoprotein, triglycerides, blood pressure, and low-density lipoprotein), we built predictive models using Logistic Regression and Gradient Boosting Machine (GBM) techniques. The area under the receiver operating characteristic curve (AROC) was used to evaluate the discriminatory capability of these models. We used the adjusted threshold method and the class weight method to improve sensitivity – the proportion of Diabetes Mellitus patients correctly predicted by the model. We also compared these models to other learning machine techniques such as Decision Tree and Random Forest.

**Results:**

The AROC for the proposed GBM model is 84.7% with a sensitivity of 71.6% and the AROC for the proposed Logistic Regression model is 84.0% with a sensitivity of 73.4%. The GBM and Logistic Regression models perform better than the Random Forest and Decision Tree models.

**Conclusions:**

The ability of our model to predict patients with Diabetes using some commonly used lab results is high with satisfactory sensitivity. These models can be built into an online computer program to help physicians in predicting patients with future occurrence of diabetes and providing necessary preventive interventions. The model is developed and validated on the Canadian population which is more specific and powerful to apply on Canadian patients than existing models developed from US or other populations. Fasting blood glucose, body mass index, high-density lipoprotein, and triglycerides were the most important predictors in these models.

## Background

Diabetes Mellitus (DM) is an increasingly prevalent chronic disease characterized by the body’s inability to metabolize glucose. Finding the disease at the early stage helps reduce medical costs and the risk of patients having more complicated health problems. Wilson et al. [18] developed the Framingham Diabetes Risk Scoring Model (FDRSM) to predict the risk for developing DM in middle-aged American adults (45 to 64 years of age) using Logistic Regression. The risk factors considered in this simple clinical model are parental history of DM, obesity, high blood pressure, low levels of high-density lipoprotein cholesterol, elevated triglyceride levels, and impaired fasting glucose. The number of subjects in the sample was 3140 and the area under the receiver operating characteristic curve (AROC) was reported to be 85.0%. The performance of this algorithm was evaluated in a Canadian population by Mashayekhi et al. [11] using the same predictors as Wilson et al. [18] with the exception of parental history of DM. The number of subjects in the sample was 4403 and the reported AROC was 78.6%.

Data mining techniques have been widely used in DM studies to explore the risk factors for DM [5, 6, 8, 12]. Machine learning methods, such as logistic regression, artificial neural network, and decision tree were used by Meng et al. [12] to predict DM and pre-diabetes. The data included 735 patients who had DM or pre-diabetes and 752 who are healthy from Guangzhou, China. The accuracy was reported to be 77.87% using a decision tree model; 76.13% using a logistic regression model; and 73.23% using the Artificial Neural Network (ANN) procedure. Other machine learning methods, such as Random Forest, Support Vector Machines (SVM), k-nearest Neighbors (KNN), and the naïve Bayes have also been used as in [6–8, 10, 11, 21]. Sisodia, D. and Sisodia, D.S [17]. recently used three classification algorithms: Naïve Bayes, Decision Tree, and SVM, to detect DM. Their results showed that Naïve Bayes algorithm works better than the other two algorithms.

In this article, we present predictive models using Gradient Boosting Machine and Logistic Regression techniques to predict the probability of patients having DM based on their demographic information and laboratory results from their visits to medical facilities. We also compare these methods with other widely used machine learning techniques such as Rpart and Random Forest. The MLR (Machine Learning in R) package in R [2] was used to develop all the models.

## Methods

The data used in this research were obtained from CPCSSN (www.cpcssn.ca). The case definition for diabetes is described in [19]. “Diabetes includes diabetes mellitus type 1 and type 2, controlled or uncontrolled, and excludes gestational diabetes, chemically induced (secondary) diabetes, neonatal diabetes, polycystic ovarian syndrome, hyperglycemia, prediabetes, or similar states or conditions” (page 4 in [19]). The dataset was generated as follows: 1) Every blood pressure reading (over 6 million) were pulled into a table for all patients over the age of 17 along with the patient ID, their age on the date of the exam and their sex. 2) For each blood pressure reading, we joined the following records that were closest in time, within a specific time period, based on the type of measurement: BMI ± 1 year, LDL ± 1 year, HDL ± 1 year, triglyceride (TG) ± 1 year, Fasting blood sugar (FBS) ± 1 month, HbA1c ± 3 months. 3) We removed records with missing data in any one of the columns. This left approximately 880,000 records, of which approximately 255,000 records were from patients who have diabetes. 4) Patients on insulin, who might have Type 1 diabetes, and patient on corticosteroids, which can affect blood sugar levels, were removed from the dataset, leaving 811,000 records with 235,000 from patients with DM. 5) We then curated a dataset for records of patients that preceded the onset of DM and identified those patients for whom there were at least 10 visits worth of data. For patients who had not developed DM, we removed the last year of records before the end of the database to minimize the impact of patients who might be on the verge of becoming diabetic.

There are 215,544 records pertaining to patient visits in the dataset. The outcome variable is Diabetes Mellitus which is encoded a binary variable, with category 0 indicating patients with no DM and category 1 indicating patients with DM. The predictors of interest are: Sex, Age (Age at examination date), BMI (Body Mass Index), TG (Triglycerides), FBS (Fasting Blood Sugar), sBP (Systolic Blood Pressure), HDL (High Density Lipoprotein), and LDL (Low Density Lipoprotein). Since a patient may have multiple records representing their multiple visits to medical facilities, we took each patient’s last visit to obtain a dataset with 13,317 patients. In the exploratory data analysis step, we found some extreme values in BMI and TG, and thereafter, excluded these values to obtain a final analysis dataset with 13,309 patients.

About 20.9% of the patients in this sample have DM. 40% of the patients are male and about 60% are female (Additional file 1: Table S1). The age of the patients in this dataset ranges from 18 to 90 years with a median of around 64 years. Age is also encoded as a categorical variable represented by the four categories: Young, Middle-Aged, Senior, and Elderly. About 44.6% of patients are middle-aged, between 40 and 64 years old; 47.8% are senior, between 65 and 84; 4.8% are elderly who are older than 85; and 2.9% are younger than 40 years old. Body mass index was calculated by dividing the patient’s weight (in kilograms) by the patient’s height (in meters) squared. The body mass index ranges from 11.2 to 70 with a median of 28.9. The distributions of BMI, FBS, HDL and TG are all right-skewed (Additional file 2: Figure S1).

Table 1 shows that the medians of BMI, FBS, and TG of the group of patients with DM are higher than those of the group of patients with no DM; the median HDL is higher for the group of patients with no DM meanwhile the median LDL, median sBP, and the median Age are similar.

**Table 1:** Comparing the median of continuous variables between DM and No DM groups

Group	BMI	FBS	HDL	TG	LDL	sBP	Age
DM	31.16	6.10	1.20	1.56	2.71	130	64.00
No DM	28.32	5.20	1.40	1.24	2.74	130	66.00

The correlation matrix of the continuous variables (Age, BMI, TG, FBS, sBP, HDL, LDL) shows no remarkable correlation among the variables, except for a moderate negative correlation of − 0.39 between HDL and TG.

Gradient Boosting Machine is a powerful machine-learning technique that has shown considerable success in a wide range of practical applications [14]. In this research study, we used Logistic Regression and Gradient Boosting Machine techniques in the MLR package in R to build predictive models. We then compared these methods to two other modern machine-learning techniques which are Decision Tree Rpart and Random Forest.

### Procedure

We first created a training dataset by randomly choosing 80% of all patients in the dataset and created a test dataset with the remaining 20% of patients. The training dataset has 10,647 patients and the test dataset has 2662 patients. We used the training dataset to train the model and used the test dataset to evaluate how well the model performs based on an unseen dataset. Using the training dataset and the 10-fold cross-validation method, we tuned the model hyperparameters to obtain the set of optimal hyperparameters that yields the highest area under the receiver operating characteristic curve (AROC). (Please see Additional file 3 for our model tuning process).

Since the dataset is imbalanced with only 20.9% of the patients in the DM group, we used different misclassification costs to find the optimal threshold (or the cut off value) for the DM class (i.e., Diabetes Mellitus =1). In the tuning threshold approach, we set up a matrix of misclassification costs in which the diagonal elements are zero and the ratio of the cost of a false negative to the cost of a false positive is 3 to 1. We validated the model with the optimal hyperparameters using a 10-fold cross validation. In this step, we measured both AROC values and the misclassification costs. We tuned the threshold for the positive class (Diabetes = 1) by choosing the threshold that yields the lowest expected misclassification cost. We obtained our final model by fitting the model with the optimal set of hyperparameters on the entire training dataset. Finally, using the optimal threshold we evaluated the performance of the final model on the test dataset. Sensitivity was calculated by dividing the model-predicted number of DM patients by the observed number of DM patients. Specificity was calculated by dividing the model-predicted number of No DM patients by the observed number of No DM patients. The misclassification rate is the number of incorrectly classified patients divided by the total number of patients.

## Results

The optimal set of hyperparameters we obtained for this GBM model is as follows: the number of iterations (n.trees) is 257; the interaction depth (interaction.depth) is 2; the minimum number of observations in the terminal nodes (n.minobsinnode) is 75; the shrinkage rate (shrinkage) is 0.126. Since the outcome variable is a binary variable, we used the Bernoulli loss function and tree-based learners in this GBM model. Using the cross-validation method to validate this model, we obtained AROC values ranging from 81.6 to 85.0% with an average AROC of 83.6%, indicating a high reliability of the method. The optimal threshold for the DM class using the misclassification cost matrix method is 0.24. We also used the train/test split method to validate this model and obtained similar results with average AROC of 83.3%.

When testing the model on the test dataset we obtained the following results: the AROC is 84.7%; the misclassification rate is 18.9%; the sensitivity is 71.6% and the specificity is 83.7%. We observed that there is a trade off between the sensitivity and the misclassification rate. Using a default threshold of 0.5, the misclassification rate for the GBM model was 15%; the sensitivity was low at 48.3%; the specificity was 95.2%; and the AROC remained the same at 84.7%.

For our Logistic Regression model, the AROC was 84.0%; the misclassification rate was 19.6%; the sensitivity was 73.4% and the specificity was 82.3%. The optimal threshold was estimated to be 0.24 and Age was treated as a categorical variable in this model. We validated this model using the cross-validation method and obtained AROC values ranging from 80.6 to 85.7% with an average AROC of 83.2%. Fasting blood glucose, high-density lipoprotein, body mass index, and triglycerides were very significant predictors in this model (*P* < 0.0001). Interestingly, based on this sample data, we found that age was also a significant factor (Table 2); elderly and senior patients significantly have lower chance of having DM than the middle-aged patients, given that all other factors are kept the same. Checking the model assumptions, we found no severe collinearity; all variables had a variance inflation factor (VIF) values less than 1.5. Variables FBS, SBP, TG, and BMI were all strongly linearly associated with the DM outcome on the logit scale. With respect to standardized residuals, there were 9 outliers ranging from 3.1 to 3.4. Since the number of potential influential observations was not large, all patients were kept in the dataset.

**Table 2:** Predictors associated with the logistic regression model

Variables	Estimated coefficient	Odds ratio	95% CI for odds ratio	*P* Value
Intercept	−11.816			< 0.0001
Age				
Middle-Aged (40–64)	(Reference)	1.000		
Elderly (85–90)	−0.829	0.436	(0.31, 0.61)	< 0.0001
Senior (65–84)	−0.127	0.881	(0.78, 0.99)	0.036
Young (< 40)	0.238	1.269	(0.90, 1.79)	0.170
Male	−0.250	0.779	(0.69, 0.88)	< 0.0001
FBS	1.963	7.122	(6.45, 7.87)	< 0.0001
BMI	0.023	1.024	(1.01, 1.03)	< 0.0001
HDL	−0.894	0.409	(0.34, 0.49)	< 0.0001
TG	0.158	1.171	(1.09, 1.26)	< 0.0001
sBP	−0.001	0.999	(0.96, 1.00)	0.560
LDL	−0.011	0.990	(0.93, 1.05)	0.740

**Fig. 1:**
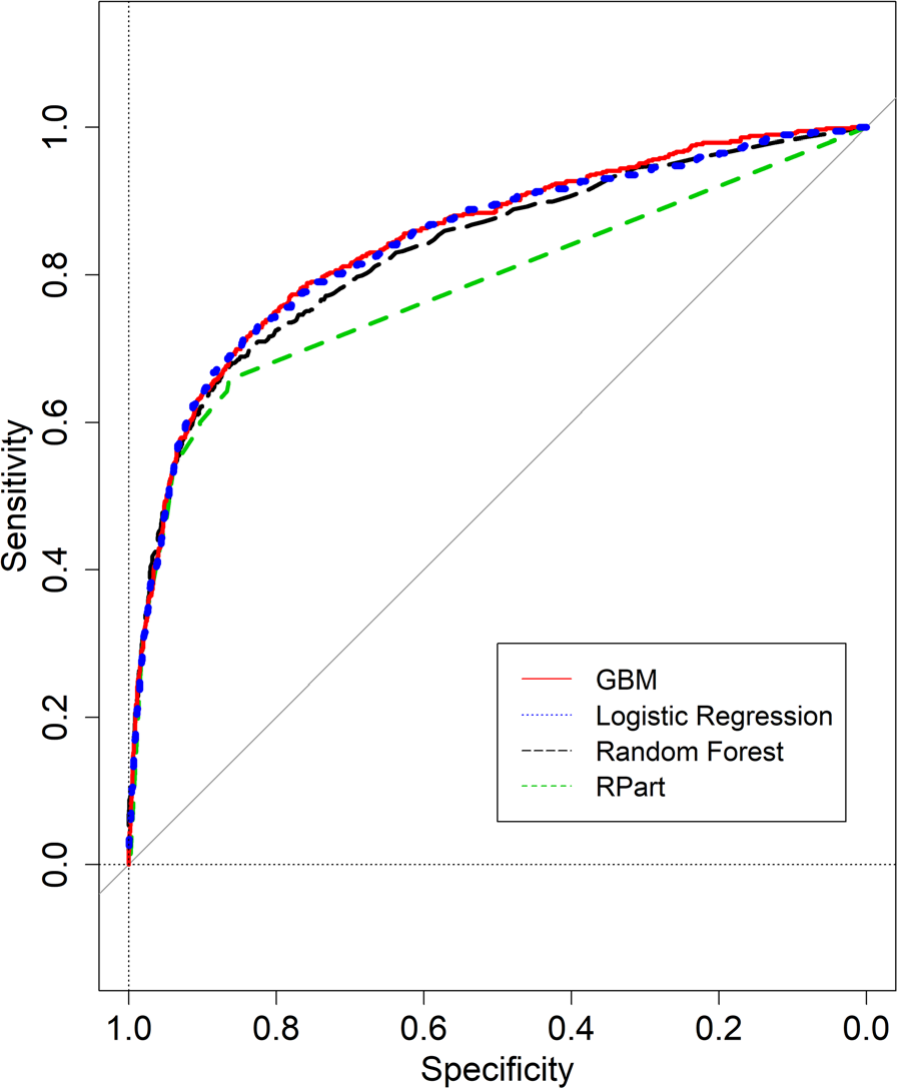
Information gain measure from predictors

Based on the information gain criterion which measures the amount of information gained by each predictor, we also found that fasting blood glucose is the most important predictor, followed by high-density lipoprotein, body mass index, and triglycerides; then age, sex, blood pressure, and low-density lipoprotein (Fig. 1).

To compare the performance of the obtained Logistic Regression and GBM models with other machine-learning techniques, we used the same training dataset, test dataset, and procedure on the Rpart and Random Forest techniques. The AROC values from the models are presented in Table 3.

**Table 3:** Comparing the AROC values with other machine-learning techniques

Model	Area under the ROC curve, AROC
GBM	84.7%
LOGISTIC REGRESSION	84.0%
RANDOM FOREST	83.4%
RPART	78.2%

The results in Table 3 show that the GBM model performs the best based on highest AROC value, followed by the Logistic Regression model and the Random Forest model. The Rpart model gives the lowest AROC value at 78.2%.

**Fig. 2:**
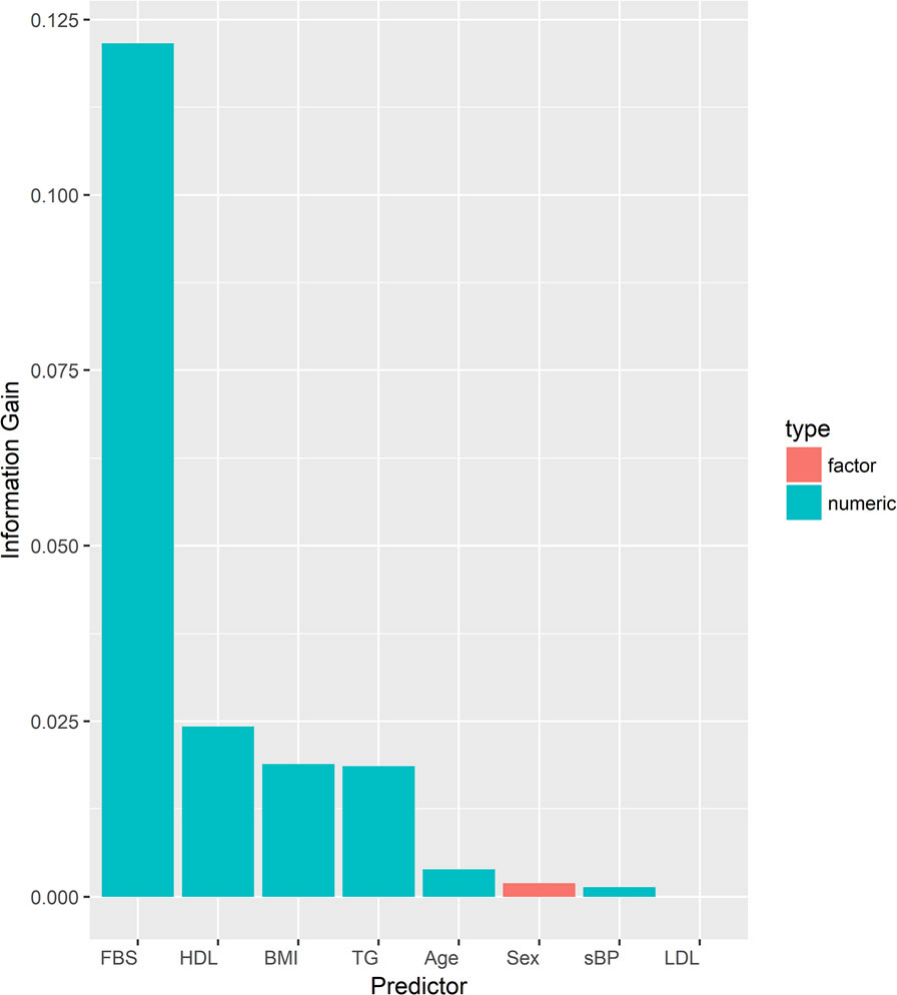
Receiver operating curves for the Rpart, random forest, logistic regression, and GBM models

Figure 2 illustrates the Receiver Operating Curves (ROC) curves of the four models.

The confusion matrices for these four models are presented in Additional file 1: Tables S2, S3, S4 and S5.

Our models can be implemented in practice. For the Logistic Regression model, we outline an algorithm for estimating the risk of DM. sBP and LDL were excluded from this model as their contributions were not statistically significant.

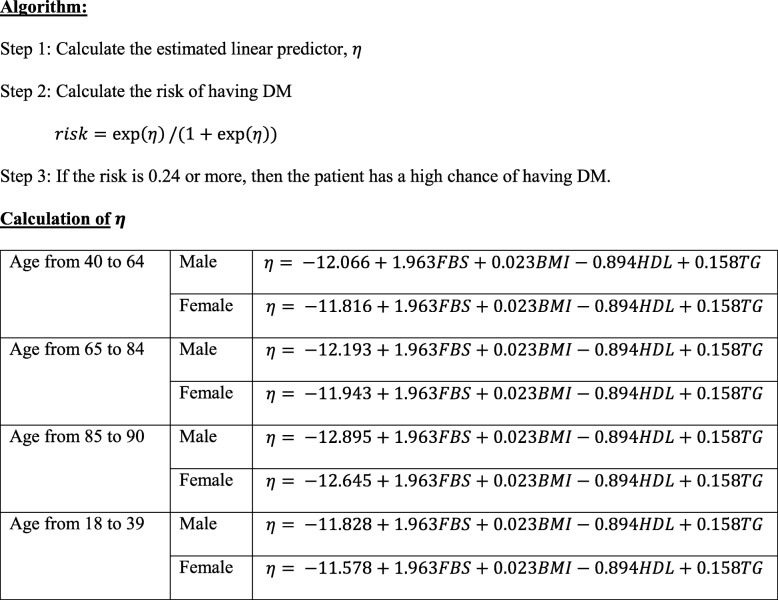



For the GBM model, it is more difficult to display the equations explicitly. However, it is feasible to set up an online real-time DM risk predictor program so that a patients’ risk of developing DM can be reported when the patient’s predictor values are entered. The trained GBM model can be saved in the Predictive Model Markup Language (PMML) format, which is an XML-based format, using the package r2pmml in R. Thereafter, the model can be deployed to make predictions using a Java platform (Scoruby and Goscore packages) or the Yellowfin platform.

To compare the performance of the four models, we conducted 10-fold cross validation on the whole dataset with the following steps:

1. Divide data set into 10 parts. Use 9 parts as training data set and the last part as the testing data set.
2. Train the four 4 models on the training data set.
3. Measure AROC for each model based on the testing data set
4. Repeat for all 10 folds

Shuffle the whole data set and repeat the above procedure 2 more times.

Based on 30 values of AROC obtained for each model (with age is treated as a continuous variable), we estimated the mean of their AROC values as shown in Table 4.

**Table 4:** Mean of AROC for the four models from the cross-validation results

	Mean
GBM	83.9%
Logistic Regression	83.5%
Random Forest	83.0%
Rpart	77.1%

**Fig. 3:**
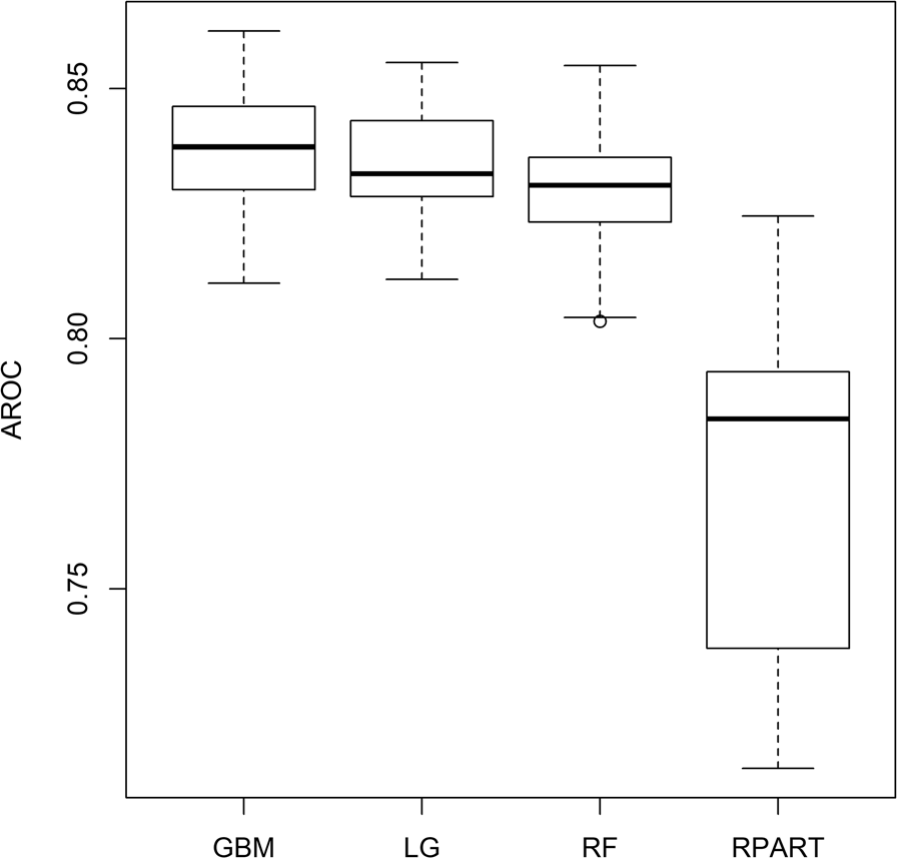
Box plot: comparing the AROC of the four models in the cross-validation results

We also created a box plot to compare the AROC values of the four models (Fig. 3).

The box plot shows that the medians of AROC values for GBM, Logistic Regression and Random Forest are quite close to each other and they are all greater than that of the Rpart model.

Due to the independence and normality assumptions of the t-test, it may not be safe to use the paired t-test for testing equality between the mean AROC values for any two models based on the AROC values we obtained. Therefore, to estimate the consistency of the predictive power for each model, we used the DeLong test [3] to find the standard deviation and the 95% confidence interval for the AROC value of each model. We also used the DeLong method to compare the AROC values of two correlated ROC curves. For each pair, we wanted to test the equality of AROCs of two ROC curves and whether the AROC value of the first mode is significantly greater than that of the second model. The DeLong method is a nonparametric method that was implemented in pROC package in R [20]. The obtained results are presented in Tables 5 and 6.

**Table 5:** AROC, standard deviation, and 95% confidence interval of AROC for the four models using the DeLong method

	AROC	Standard deviation	95% CI
GBM	84.5%	0.97%	(82.6, 86.4)
Logistic Regression	84.1%	1.01%	(82.1, 86.1)
Random Forest	83.2%	1.05%	(81.1, 85.2)
Rpart	78.1%	1.10%	(76.0, 80.3)

**Table 6:** Paired one-sided DeLong test to compare the AROC values of the four models

Test name	z-statistic	*p*-value
GBM vs. Logistic Regression	1.392	0.081
GBM vs. Random Forest	3.885	5.13e-05
GBM vs. Rpart	8.914	2.20e-16
Logistic Regression vs. Random Forest	2.038	0.021
Logistic Regression vs. Rpart	8.006	5.95e-16
Random Forest vs. Rpart	7.028	1.05e-12

The standard deviations are small and the confidence intervals are not wide. This indicates that the values of AROC of the four models are consistent.

These results show that the AROC value of the GBM model is significantly greater than that of Random Forest, and Rpart models (*P* < 0.001), but not significantly greater than that of Logistic Regression model (*P* > 0.05). The Logistic Regression model also has an AROC value greater than that of Random Forest and of Rpart. The AROC of Random Forest model is significantly greater than that of Rpart model, as well. We also noted that the comparison of the tests are statistically significant but this relative performance may be restricted to the specific population and data we are dealing with.

To see how our models work on a different data set, we used Pima Indians Dataset which is a publicly available [15]. All patients in this data set are females at least 21 years old of Pima Indian heritage. There are 768 observations with 9 variables as followings: Pregnant, number of times pregnant; Glucose, plasma glucose concentration (glucose tolerance test); BP, diastolic blood pressure (mm/Hg); Thickness (triceps skin fold thickness (mm)); Insulin (2-Hour serum insulin (mu U/ml); BMI (body mass index (weight in kg/(height in m) squared)); Pedigree (diabetes pedigree function); Age (Age of the patients in years); Diabetes (binary variable with 1 for Diabetes and 0 for No Diabetes).

When working on this data set, we noticed that there are many rows with missing data and the missing values in Glucose, BP, Thickness, and BMI are labeled as 0. For example, about 48.7% of Insulin values are missing. For purpose of validating our methods, we chose not to impute the data but excluded all rows with missing values. There are 392 observations left in the working data set in which 130 patients with diabetes and 262 without diabetes. We applied our methods on this dataset to predict whether or not a patient has diabetes. We also divided the PIMA data set into the training data set (80% of the observations) and the testing data set (20% of the observations). We trained the four models on the training data set and validate the models on the testing data set. On the testing data set, we obtained the AROC of 84.7% for GBM model, 88.0% for Logistic Regression Model, 87.1% for Random Forest Model, and 77.0% for Rpart model (Additional file 1: Table S8).

We also conducted 10-fold cross-validation and repeated the procedure for two more times.

Here are our results based on the 30 AROC values from the cross-validation results conducted on the PIMA Indian data set.

The results we obtained for this data set are quite consistent with what we observed in our main data set (Table 7). Based on these results, GBM, Logistic Regression, and Random Forest are comparable and they all give higher mean AROC than that of the Rpart model on the testing data set. We also created a box plot to compare the sampling distributions of the AROC values for the four models.

**Table 7:** Comparing the AROC values of the four models using PIMA Indian data set

	Mean
GBM	85.1%
Logistic Regression	84.6%
Random Forest	85.5%
Rpart	80.5%

**Fig. 4:**
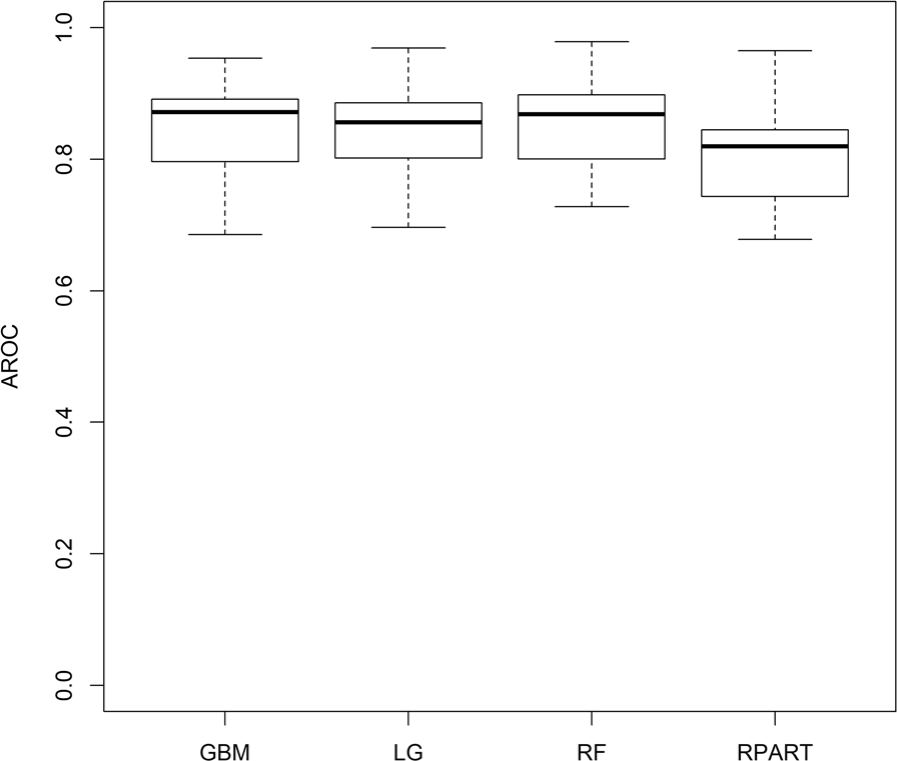
Box plot of AROC values for the Rpart, random forest, logistic regression, and GBM models applied to PIMA Indian data set

The box plot (Fig. 4) shows that the variability in the AROC values of GBM, Logistic Regression, and Random Forest are quite the same and less than that of the Rpart model.

## Discussion

In this research study, we used the Logistic Regression and GBM machine learning techniques to build a model to predict the probability that a patient develops DM based on their personal information and recent laboratory results. We also compared these models to other machine learning models to see that the Logistic Regression and GBM models perform best and give highest AROC values.

During the analysis, we also used the class weight method for our imbalanced dataset. We first tuned the class weight for the DM class to find the optimal class weight that minimized the average classification cost. We found that the optimal class weight for the GBM model is 3 and the optimal class weight for the Logistic Regression is 3.5. These optimal class weights are then incorporated into the model during the training process. We obtained similar results for GBM, Logistic Regression, and Random Forest model. However, the Decision Tree Rpart model gives a higher AROC at 81.8% compared to 78.2% when the threshold adjustment method was used (Additional file 1: Table S6). We also applied a natural logarithmic transformation on the continuous variables, however, this did not improve AROC and sensitivity.

Compared to the simple clinical model presented by Wilson et al. [18], the AROC value from our GBM model was very similar. The AROC value of our Logistic Regression model was lower, given the fact that the parental history of the disease was not available in our sample data. We also note that the characteristics of the sample data used in this study were not the same as the ones used by Wilson et al. [18]. For example, the age of the patients in our dataset ranges from 18 to 90, while the patients studied by Wilson et al. [18] ranges from 45 to 64. Schmid et al. [16] conducted a study on Swiss patients to compare different score systems used to estimate the risk of developing type 2 diabetes such as the 9-year risk score from Balkau et al. [1], the Finnish Diabetes Risk Score (FINDRISC) [13], the prevalent undiagnosed diabetes risk score from Griffin et al. [4], 10-year-risk scores from Kahn et al. [9], 8-year risk score from Wilson et al. [18], and the risk score from the Swiss Diabetes Association. Their results indicated that the risk for developing type 2 diabetes varies considerably among the scoring systems studied. They also recommended that different risk-scoring systems should be validated for each population considered to adequately prevent type 2 diabetes. These scoring systems all include the parental history of diabetes factor and the AROC values reported in these scoring systems range from 71 to 86%. Mashayekhi et al. [11] had previously applied Wilson’s simple clinical model to the Canadian population. Comparing our results to the results reported by Mashayekhi et al., the AROC values suggest that our GBM and Logistic Regression models perform better with respect to predictive ability. Using the same continuous predictors from the simple clinical model with the exception of parental history of diabetes, we also obtained an AROC of 83.8% for the Logistic Regression model on the test dataset.

## Conclusion

The main contribution of our research study was proposing two predictive models using machine-learning techniques, Gradient Boosting Machine and Logistic Regression, in order to identify patients with high risk of developing DM. We applied both the classical statistical model and modern learning-machine techniques to our sample dataset. We dealt with the issue of imbalanced data using the adjusted-threshold method and class weight method. The ability to detect patients with DM using our models is high with fair sensitivity. These predictive models are developed and validated on Canadian population reflecting the risk patterns of DM among Canadian patients. These models can be set up in a computer program online to help physicians in assessing Canadian patients’ risk of developing Diabetes Mellitus.

## Supplementary information

**Additional file 1:** **Table S1.** Summary of characteristics of patients in the dataset. **Table S2.** Confusion Matrix for the Gradient Boosting Machine (GBM) model with the threshold of 0.24. **Table S3.** Confusion Matrix for the Logistic Regression model with the threshold of 0.24. **Table S4.** Confusion Matrix for the Random Forest model with the threshold of 0.24. **Table S5.** Confusion Matrix for the Rpart model with the threshold of 0.18. **Table S6.** Comparing the AROC with other machine-learning techniques using the class weight method. **Table S7.** Sensitivity, Specificity, Misclassification Rate, and AROC values of the four models on the studied data set. **Table S8.** Sensitivity, Specificity, Misclassification Rate, and AROC values of the four models on the PIMA Indians data set.

**Additional file 2:** **Figure S1**. Scatter Plot Matrix of Continuous Variables.

**Additional file 3.** Description of the model tuning process. Model Diagram.

## Data Availability

The data that support the findings of this study are available from CPCSSN (www.cpcssn.ca) but restrictions apply to the availability of these data, which were used under license for the current study, and so are not publicly available. Data are however available from the authors upon reasonable request and with permission of CPCSSN.

## Abbreviations

AROC: Area under the receiver operating characteristics curve
BMI: Body mass index
DM: Diabetes mellitus
FBS: Fasting blood sugar
GBM: Gradient boosting machine
HDL: High density lipoprotein
LDL: Low density lipoprotein
sBP: Systolic blood pressure
TG: Triglycerides
