# Supplementary material for: Predictive models for diabetes mellitus using machine learning techniques
Source: BMC Endocr Disord. 2019 Oct 15;19:101. doi: 10.1186/s12902-019-0436-6 (PMC6794897; doi:10.1186/s12902-019-0436-6)
Supplement: Supplementary file 1 — Additional file 1: Table S1. Summary of characteristics of patients in the dataset. Table S2. Confusion Matrix for the Gradient Boosting Machine (GBM) model with the threshold of 0.24. Table S3. Confusion Matrix for the Logistic Regression model with the threshold of 0.24. Table S4. Confusion Matrix for the Random Forest model with the threshold of 0.24. Table S5. Confusion Matrix for the Rpart model with the threshold of 0.18. Table S6. Comparing the AROC with other machine-learning techniques using the class weight method. Table S7. Sensitivity, Specificity, Misclassification Rate, and AROC values of the four models on the studied data set. Table S8. Sensitivity, Specificity, Misclassification Rate, and AROC values of the four models on the PIMA Indians data set. [file 12902_2019_436_MOESM1_ESM.docx]

**Additional File 1**

**Table S1**

Summary of characteristics of patients in the data set.

| **Factor** | **Findings** |
| --- | --- |
| Number of subjects | 13309 |
| Diabetes, *n (%)* |  |
| Yes (1) | 2780 (21%) |
| No (0) | 10529 (79%) |
| Sex, *n (%)* |  |
| Male | 5314 (40%) |
| Female | 7995 (60%) |
| Variables, *Mean (SD)* |  |
| Age | 64.8 (12.62) |
| BMI | 29.9 (6.46) |
| HDL | 1.41 (0.40) |
| LDL | 2.79 (0.91) |
| TG | 1.48 (0.77) |
| sBP | 130 (15.70) |
| FBS | 5.44 (0.67) |

**Table S2**

Confusion Matrix for the Gradient Boosting Machine (GBM) model with the threshold

of 0.24.

|  | **True No DM** | **True DM** |
| --- | --- | --- |
| **Predicted No DM** | 1745 | 164 |
| **Predicted DM** | 339 | 414 |

**Table S3**

Confusion Matrix for the Logistic Regression model with the threshold of 0.24.

|  | **True No DM** | **True DM** |
| --- | --- | --- |
| **Predicted No DM** | 1715 | 154 |
| **Predicted DM** | 369 | 424 |

**Table S4**

Confusion Matrix for the Random Forest model with the threshold of 0.24.

|  | **True No DM** | **True DM** |
| --- | --- | --- |
| **Predicted No DM** | 1758 | 182 |
| **Predicted DM** | 326 | 396 |

**Table S5**

Confusion Matrix for the Rpart model with the threshold of 0.18.

|  | **True No DM** | **True DM** |
| --- | --- | --- |
| **Predicted No DM** | 1807 | 200 |
| **Predicted DM** | 277 | 378 |

**Table S6**

Comparing the AROC with other machine-learning techniques using the class weight method.

| **Model** | **Area Under the ROC Curve, AROC** |
| --- | --- |
| GBM, w* = 3 | 84.1% |
| RPART, w = 2.75 | 81.8% |
| RANDOM FOREST, w = 1.75 | 82.9% |
| LOGISTIC REGRESSION, w = 3.25 | 84.1% |

* w is the class weight for the DM class.

**Table S7**

Sensitivity, Specificity, Misclassification Rate, and AROC values of the four models on the studied data set using cutoff value of 0.24 for GBM, Logistic Regression, Random Forest, and a cutoff value of 0.18 for Rpart model.

| **Model** | **AROC** | **Misclassification Rate** | **Sensitivity** | **Specificity** |
| --- | --- | --- | --- | --- |
| GBM | 84.7% | 18.9% | 71.6% | 83.7% |
| LOGISTIC REGRESSION | 84.0% | 19.6% | 73.4% | 82.3% |
| RANDOM FOREST | 83.4% | 19.1% | 68.5% | 84.4% |
| RPART | 78.2% | 17.9% | 65.4% | 86.7% |

**Table S8**

Sensitivity, Specificity, Misclassification Rate, and AROC values of the four models on the PIMA Indians data set using cutoff value of 0.3 for GBM, Logistic Regression, Random Forest, and a cutoff value of 0.18 for Rpart model.

| **Model** | **AROC** | **Misclassification Rate** | **Sensitivity** | **Specificity** |
| --- | --- | --- | --- | --- |
| GBM | 84.7% | 21.5% | 81.5% | 76.9% |
| LOGISTIC REGRESSION | 88.0% | 20.3% | 81.5% | 78.8% |
| RANDOM FOREST | 87.1% | 19.0% | 68.5% | 80.8% |
| RPART | 77.0% | 21.5% | 66.7% | 84.6% |
