# Supplementary figures and images for: Predictive models for diabetes mellitus using machine learning techniques
Source: BMC Endocr Disord. 2019 Oct 15;19:101. doi: 10.1186/s12902-019-0436-6 (PMC6794897; doi:10.1186/s12902-019-0436-6)

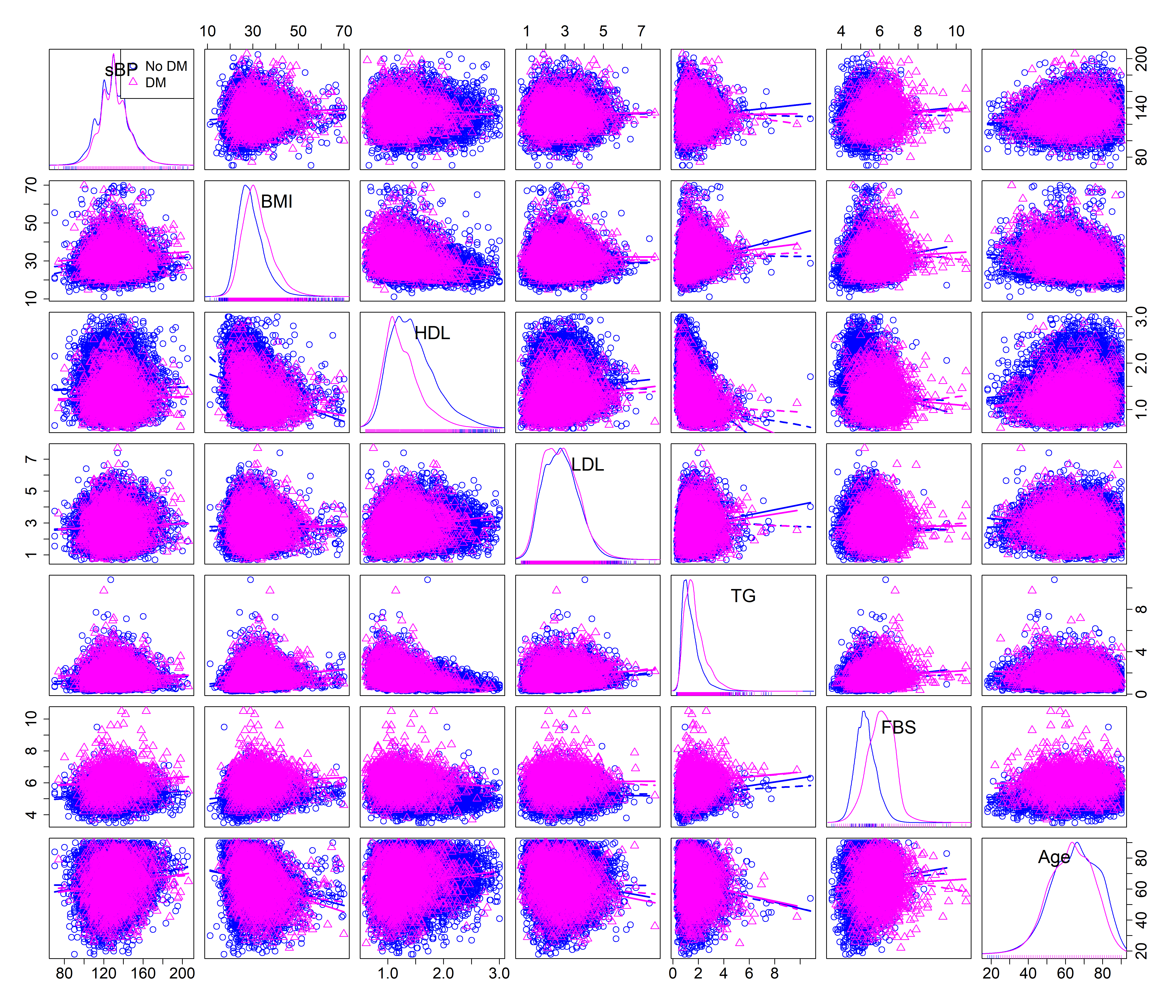

Supplement: Supplementary file 2 — Additional file 2: Figure S1. Scatter Plot Matrix of Continuous Variables. [file 12902_2019_436_MOESM2_ESM.tiff]
