# Supplementary material for: Predictive models for diabetes mellitus using machine learning techniques
Source: BMC Endocr Disord. 2019 Oct 15;19:101. doi: 10.1186/s12902-019-0436-6 (PMC6794897; doi:10.1186/s12902-019-0436-6)
Supplement: Supplementary file 3 — Additional file 3. Description of the model tuning process. Model Diagram. [file 12902_2019_436_MOESM3_ESM.docx]

**Additional file 3**

**Description of the tuning process for each model:**

The training dataset as described in the manuscript was used in the tuning process.

1. **GBM model:**

**The parameter search space**: We create a search space by defining a parameter space for hyperparameters as follows: the number of trees (n.trees) is an integer ranging from 200 to 600; the depth of tree (interaction.depth) is from 2 to 6; the minimum number of observations in the terminal nodes (n.minobsinnode) is from 30 to 80; and learning rate (shrinkage) is from 0.01 to 0.3.

**Tuning method**: We perform random search on the parameter space with the number of iterations of 100.

**Evaluation method**: We use 10-fold cross validation and use AROC as the performance measure. That is, for each iteration, a 10-fold cross validation is conducted and the average AROC is reported. Then from the 100 iterations, the model with the highest average AROC is chosen.

The tuning results are as follows: the number of iterations (n.trees) is 257; the interaction depth (interaction.depth) is 2; the minimum number of observations in the terminal nodes (n.minobsinnode) is 75; the shrinkage rate (shrinkage) is 0.126.

1. **Logistic Regression model:**

There are no hyperparameters for the Logistic Regression models so we do not use the tuning process for Logistic Regression models.

1. **Random Forest model:**

**Parameter search space**: the number of trees to grow (ntree) is from 80 to 500; the number of variables should be selected at a node split (mtry) is an integer ranging from 3 to 6; the number of observations at terminal nodes (nodesize) is from 20 to 50.

**Tuning method**: We perform random search on the parameter space with the number of iterations of 100.

**Evaluation method**: We use 10-fold cross validation and use AROC as the performance measure. Then from the 100 iterations, the model with the highest average AROC is chosen.

The tuning results are as follows: the number of trees to grow (ntree) is 407; the number of variables should be selected at a node split (mtry) is 3; the number of observations at terminal nodes (nodesize) is 22.

1. **Decision Tree model:**

**Parameter search space**: the smallest number of observations in the parent node that could be split further (minsplit) is from 30 to 50; the smallest number of observations that are allowed in a terminal node (minbucket) is an integer ranging from 10 to 50; depth of tree (maxdepth) can be 8, 12, or 16; the complexity parameter (cp) is from 0.001 to 0.2.

**Tuning method**: We create a grid for the parameter space with resolution of 10.

**Evaluation method**: We use 10-fold cross validation and use AROC as the performance measure. Then from the 3000 iterations, the model with the highest average AROC is chosen.

The tuning results are as follows: The smallest number of observations in the parent node that could be split further (minsplit) is 41; the smallest number of observations that are allowed in a terminal node (minbucket) is 19; the depth of tree (maxdepth) is 8; the complexity parameter (cp) is 0.001.

**The model diagram:**

Run the developed models on the Testing Set

Testing Set

Compare the four models based on AROC

Training Set

Develop four models:

GBM, Logistic Regression,

Random Forest, Decision Tree

Data Preparation
